# SARS-CoV-2 introductions and early dynamics of the epidemic in Portugal

**DOI:** 10.1038/s43856-022-00072-0

**Published:** 2022-01-28

**Authors:** Vítor Borges, Joana Isidro, Nídia Sequeira Trovão, Sílvia Duarte, Helena Cortes-Martins, Hugo Martiniano, Isabel Gordo, Ricardo Leite, Luís Vieira, Portuguese network for SARS-CoV-2 genomics (Consortium), Agostinho José S. Lira, Aida M. Sousa Fernandes, Alexandra Estrada, Alexandra Nunes, Alfredo Rodrigues, Ana Caldas, Ana Constança, Ana Margarida Henriques, Ana Miguel Matos, Ana Oliveira, Ana Paula Dias, Ana Pelerito, Ana Rita Couto, Anabela Vilares, António Albuquerque, Baltazar Nunes, Bruna R. Gouveia, Carina de Fátima Rodrigues, Carla Feliciano, Carla Roque, Carlos Cardoso, Carlos Sousa, Cathy Paulino, Célia Rodrigues Bettencourt, Claudia C. Branco, Cláudia Nunes dos Santos, Conceição Godinho, Constantino P. Caetano, Cristina Correia, Cristina Toscano, Cristina Veríssimo, Daniela Silva, Diana Patrícia Pinto da Silva, Eliana Costa, Elizabeth Pádua, Fátima Martins, Fátima Vale, Fernanda Vilarinho, Fernando Branca, Filomena Caldeira, Filomena Lacerda, Francisca Rocha, Graça Andrade, Helena Ribeiro, Helena Rodrigues, Herberto Jesus, Hugo Sousa, Idalina Ferreira, Inês Baldaque, Inês Costa, Inês Gomes, Inna Slobidnyk, Isabel Albergaria, Isabel Dias, Isabel Fernandes, Isabel Lopes de Carvalho, Ivone Água-Doce, Jácome Bruges Armas, Joana Ramos, João Carlos Sousa, João Costa, João Dias, João Rodrigues, João Sobral, Jorge Machado, Jorge Meneses, José Alves, José Vicente Constantino, Laura Brum, Leonor Silveira, Líbia Zé-Zé, Lidia Santos, Ludivina Freitas, Luís Silva, Luisa Mota-Vieira, Lurdes Lopes, Lurdes Monteiro, Márcia Faria, Margarida Farinha, Margarida Vaz, Maria Alice Pinto, Maria Ana Pessanha, Maria Beatriz Tomaz, Maria Calle Vellés, Maria da Graça Maciel de Soveral, Maria Helena Ramos, Maria Isabel Veiga, Maria João Gargate, Maria João Peres, Maria José Borrego, Maria Matos Figueiredo, Mariana Martins, Mariana Viana, Maurício Melim, Miguel Babarro Jorreto, Miguel Fevereiro, Miguel Pinheiro, Mónica Oleastro, Nair Seixas, Nelson Ventura, Nuno Verdasca, Olga Costa, Patrícia Barros, Patricia Fonseca, Patricia Miguel, Paula Bajanca-Lavado, Paula Branquinho, Paula Palminha, Paula Soares, Paula Valente, Paulo Leandro, Paulo Pereira, Pedro Cardoso, Pedro Pechirra, Pedro Ramos, Raquel Neves, Raquel Rocha, Raquel Rodrigues, Raquel Sabino, Regina Sá, Ricardo Filipe Romão Ferreira, Ricardo Rodrigues, Rita C. Veloso, Rita Cordeiro, Rita Côrte-Real, Rita de Sousa, Rita Gralha, Rita Macedo, Rita Matos, Rita Rodrigues, Sandra Paulo, Sara Sousa, Sílvia Lopo, Sónia Marta Santos Magalhães, Sónia Rodrigues, Sónia Silva, Susana Ladeiro, Susana Martins, Susana Silva, Teresa Salvado, Tiago Luís, Valquíria Alves, Vera Manageiro, Raquel Guiomar, João Paulo Gomes

**Affiliations:** 1 Bioinformatics Unit, Department of Infectious Diseases, National Institute of Health Doutor Ricardo Jorge (INSA), Lisbon, Portugal; 2 Division of International Epidemiology and Population Studies, Fogarty International Center, National Institutes of Health, Bethesda, MD USA; 3 Innovation and Technology Unit, Department of Human Genetics, National Institute of Health Doutor Ricardo Jorge (INSA), Lisbon, Portugal; 4 Reference and Surveillance Unit, Department of Infectious Diseases, National Institute of Health Doutor Ricardo Jorge (INSA), Lisbon, Portugal; 5 Instituto Gulbenkian de Ciência (IGC), Oeiras, Portugal; 6 Centre for Toxicogenomics and Human Health (ToxOmics), Genetics, Oncology and Human Toxicology, Nova Medical School; Faculdade de Ciências Médicas, Universidade Nova de Lisboa, Lisbon, Portugal; 7 National Reference Laboratory for Influenza and other Respiratory Viruses, Department of Infectious Diseases, National Institute of Health Doutor Ricardo Jorge (INSA), Lisbon, Portugal; 8 Centro Hospitalar de Vila Nova de Gaia e Espinho, Vila Nova de Gaia, Portugal; 9 Laboratório Regional de Saúde Pública Drª Laura Ayres - ARS Algarve, Almancil, Portugal; 10 Serviço de Patologia Clínica, Hospital de Braga, Braga, Portugal; 11 Instituto Nacional de Saúde Dr Ricardo Jorge (INSA), Lisboa, Portugal; 12 Beatriz Godinho Saúde, Leiria, Portugal; 13 Serviço de Patologia Clínica, Centro Hospitalar Tondela-Viseu, Viseu, Portugal; 14 Serviço de Microbiologia, Centro Hospitalar do Porto, Porto, Portugal; 15 Instituto Nacional de Investigação Agrária e Veterinária, Lisboa, Portugal; 16 Laboratório de Análises Clínicas da Universidade de Coimbra, Coimbra, Portugal; 17 ACES de Baixo Vouga, Administração Regional de Saúde do Centro, Aveiro, Portugal; 18 Laboratório de Microbiologia e Biologia Molecular do Centro Hospitalar de Lisboa Ocidental, Lisboa, Portugal; 19 Serviço Especializado de Epidemiologia e Biologia Molecular, Hospital de Santo Espírito, Ilha Terceira, Açores Angra do Heroísmo, Portugal; 20 Unidade Local de Saúde de Matosinhos, Matosinhos, Portugal; 21 Interactive Technologies Institute - LARSyS, Funchal, Portugal; 22 Centro de Investigação de Montanha, Instituto Politécnico de Bragança, Bragança, Portugal; 23 Laboratório de Análises Clínicas Dr Joaquim Chaves, Lisboa, Portugal; 24 Laboratório De Biologia Molecular da Unilabs, Porto, Portugal; 25 Unidade de Genética e Patologia Moleculares, Hospital do Divino Espirito Santo de Ponta Delgada, Ilha de S. Miguel, Açores, Ponta Delgada Portugal; 26 Centro de Estudos de Doenças Crónicas, Faculdade de Ciências Médicas, Universidade Nova de Lisboa, Lisboa, Portugal; 27 Laboratório Biologia Molecular- Serviço de Patologia Clínica, Centro Hospitalar Universitário Lisboa Central, Lisboa, Portugal; 28 ALS Controlvet, Tondela, Portugal; 29 Centro Médico da Praça, São João da Madeira, Portugal; 30 Serviço de Patologia Clínica, Centro Hospitalar de Trás-os-Montes e Alto Douro, Vila Real, Portugal; 31 Serviço de Patologia Clínica, Unidade Local de Saúde da Guarda, Guarda, Portugal; 32 Hospital Espírito Santo, Évora, Portugal; 33 Laboratório Dra Helena Rodrigues, Valença, Portugal; 34 Serviço de Patologia Clínica - Hospital Dr. Nélio Mendonça - SESARAM, Funchal, Portugal; 35 Serviço de Patologia Clínica, Hospital de Vila Franca de Xira, Vila Franca de Xira, Portugal; 36 Instituto de Administração da Saúde da Madeira, Funchal, Portugal; 37 Serviço de Virologia, Instituto Português de Oncologia do Porto, Porto, Portugal; 38 Serviço de Patologia Clinica - Unidade Local de Saúde Litoral Alentejano, Santiago do Cacém, Portugal; 39 Life and Health Sciences Research Institute, School of Medicine, University of Minho, Braga, Portugal; 40 Synlab, Lisboa, Portugal; 41 Secção de Patologia Molecular, Synlab, Lisboa, Portugal; 42 Centro Hospitalar Tâmega e Sousa, Penafiel, Portugal; 43 Serviço de Imunohemoterapia, Unidade Local de Saúde do Alto Minho, Viana do Castelo, Portugal; 44 Laboratório de Imunologia e Biologia Molecular, Centro Hospitalar de Setúbal, Setúbal, Portugal; 45 Serviço de Patologia Clínica da Unidade Local de Saúde de Castelo Branco, Castelo Branco, Portugal; 46 iBiMED/Universidade de Aveiro, Aveiro, Portugal; 47 Departamento de Saúde Pública e Planeamento, ARS Alentejo, Évora, Portugal; 48 Secretaria Regional de Saúde e Proteção Civil - Governo Regional da Madeira, Funchal, Portugal; 49 Hospital Agostinho Ribeiro-Felgueiras, Felgueiras, Portugal

**Keywords:** Viral infection, SARS-CoV-2

## Abstract

**Background:**

Genomic surveillance of SARS-CoV-2 in Portugal was rapidly implemented by the National Institute of Health in the early stages of the COVID-19 epidemic, in collaboration with more than 50 laboratories distributed nationwide.

**Methods:**

By applying recent phylodynamic models that allow integration of individual-based travel history, we reconstructed and characterized the spatio-temporal dynamics of SARS-CoV-2 introductions and early dissemination in Portugal.

**Results:**

We detected at least 277 independent SARS-CoV-2 introductions, mostly from European countries (namely the United Kingdom, Spain, France, Italy, and Switzerland), which were consistent with the countries with the highest connectivity with Portugal. Although most introductions were estimated to have occurred during early March 2020, it is likely that SARS-CoV-2 was silently circulating in Portugal throughout February, before the first cases were confirmed.

**Conclusions:**

Here we conclude that the earlier implementation of measures could have minimized the number of introductions and subsequent virus expansion in Portugal. This study lays the foundation for genomic epidemiology of SARS-CoV-2 in Portugal, and highlights the need for systematic and geographically-representative genomic surveillance.

## Plain language summary

Analysing SARS-CoV-2 genetic material and how it changes over time can help us understand how the virus spreads between countries and determine the impact of control measures. In this study, we investigated SARS-CoV-2 transmission and evolution in the early stages of the COVID-19 pandemic in Portugal. In particular, we reconstructed the routes and timeliness of viral introductions into the country and assessed the relative contribution of each introduction in terms of how the epidemic evolved over time. We detected at least 277 independent introductions, mostly from European countries (namely the United Kingdom, Spain, France, Italy, and Switzerland), which were consistent with the countries with the highest connectivity with Portugal. This study reflects an unprecedented effort in the field of the infectious diseases in Portugal, highlighting the need for systematic and geographically-representative surveillance to aid public health efforts to control the virus.

## Abstract (web summary)

Borges et al. study the dynamics of SARS-CoV-2 in Portugal during 2020. The authors use phylodynamic modelling to infer patterns of SARS-CoV-2 introduction into Portugal and its subsequent spread, providing a comprehensive genomic epidemiology study of the early SARS-CoV-2 epidemic in Portugal.

## Introduction

SARS-CoV-2 (Severe Acute Respiratory Syndrome Coronavirus 2), the causative agent of COVID-19, is a novel betacoronavirus that was first reported in December 2019 in Wuhan, China^1,2^. By 29 March 2021, it had already caused more than 126 million cases and 2.7 million deaths worldwide^3,4^. In order to control the virus arrival and spread, many countries adopted rigid public health measures, including complete border closures and general lockdowns, with tremendous consequences at economic and social levels. At the early stages of an epidemic, the success of public health measures is particularly dependent on their timely implementation, which requires comprehensive diagnosis/surveillance systems that are able to efficiently trace where the virus is being introduced and circulating^5–7^. Taking advantage of the extraordinary advances in sequencing technologies, modern surveillance systems are progressively relying on genomic epidemiology as a crucial tool for outbreak investigation and for tracking virus evolution and spread^7–9^. Genomic surveillance of SARS-CoV-2 can be particularly useful to: (i) understand the contribution of “new introductions” versus “local transmission” to the number of new cases at continent/country/regional levels; (ii) evaluate the impact of non-pharmaceutical interventions on the outcomes of transmission chains; (iii) characterize the genetic variability that may negatively affect molecular diagnostic tests; (iv) monitor genetic variability affecting antigens and targets of antiviral drugs with potential impact on the development/effectiveness of prophylactic (vaccines) and therapeutic measures; and (v) investigate potential associations between genetic variants and infectious load, patient immunological status, clinical outcomes (e.g., infection duration, disease severity, etc.)^5,10^.

Acting as the National Reference Laboratory for SARS-CoV-2, the Portuguese National Institute of Health (INSA) Doutor Ricardo Jorge rapidly established a genome-based molecular surveillance strategy for SARS-CoV-2 in Portugal, setting up a large nationwide network involving more than 50 laboratories. A bilingual website (https://insaflu.insa.pt/covid19) was launched, providing updated data regarding the analysis of the SARS-CoV-2 genetic diversity and geotemporal dynamics, based on state-of-the-art methodologies for real-time tracking pathogen evolution^11,12^. Also, “situation reports” with major highlights are being released periodically to participating laboratories, national and regional public health authorities, and other stakeholders.

Despite all the advantages of genomic surveillance, the uneven geographic sampling of viral genomes can severely skew phylogeographic inferences based on discrete trait ancestral reconstruction^13^, therefore hindering the ability to accurately trace the seeding and dissemination patterns of SARS-CoV-2. The COVID-19 pandemic has been characterized by an unprecedented amount of genomic data and associated metadata, such as information on the patients’ recent movements prior to having developed any symptoms.

In the present study, we reconstruct and characterize the spatio-temporal dynamics of SARS-CoV-2 introductions and early dissemination in Portugal using newly developed phylodynamic models that allow integration of individual-based travel history, in order to obtain a more realistic reconstruction of the viral dynamics^13^. This includes inferences of the timelines of the first introductions, geographic location of ancestral lineages, and the contribution of detected introductions to the epidemic evolution.

## Methods

### Sample characterization

Samples used in this study were collected as part of the ongoing national SARS-CoV-2 laboratory surveillance conducted by INSA, Portugal, in collaboration with Instituto Gulbenkian de Ciência (IGC). SARS-CoV-2 positive samples (either clinical specimens or RNA) were provided by a nationwide network, consisting of more than 50 laboratories, that was established at the beginning of the epidemic in Portugal. Anonymized date of sample collection, date of illness onset, and travel history were provided by laboratories and Regional and National Health Authorities. Geographical data presented in this study refers to the Region (“Health Administration region”) of the patients’ residence or, when no information was available, to the Region of exposure or of the hospital/laboratory that collected/sent the sample.

### SARS-CoV-2 genome sequencing and assembly

SARS-CoV-2 positive RNA samples were subjected to genome sequencing using a whole-genome amplification strategy with tiled, multiplexed primers^14^ and the ARTIC Consortium protocol (https://artic.network/ncov-2019; https://www.protocols.io/view/ncov-2019-sequencing-protocol-bbmuik6w), with slight modifications, as previously described^15^. Analysis of sequence read data was conducted using the bioinformatics pipeline implemented in INSaFLU (https://insaflu.insa.pt/; https://github.com/INSaFLU), which is a web-based (and also locally installable) platform for amplicon-based next-generation sequencing data analysis^16^. Sequence inspection and validation was performed as previously described^15^.

### Classification by clades and lineages

We explored the diversity of INSA sequences using a variety of nomenclature strategies, namely Nextstrain (using https://clades.nextstrain.org/; 9 November 2020), GISAID (https://www.gisaid.org/; 23 July 2020) and Phylogenetic Assignment of Named Global Outbreak LINeages (cov-lineages.org) (https://pangolin.cog-uk.io/; 16 October 2020)^17^. While Nextstrain and GISAID clade nomenclatures provide a less detailed categorisation of globally circulating diversity, cov-lineages.org classification is focused on identifying highly specific lineages that are actively transmitting in the population. Classification is provided in Supplementary material (Supplementary Data 1).

### Assessment of genome sequencing by country

To assess the contribution of each country to the set of publicly available SARS-CoV-2 genomes and to determine the proportion of the number of genomes on the total number of reported COVID-19 cases (genome sampling) of a given country during the study period (until 31 March 2020), we obtained the number of cases per country from the COVID-19 Data Repository by the Center for Systems Science and Engineering (CSSE) at Johns Hopkins University (https://github.com/CSSEGISandData/COVID-19/blob/master/csse_covid_19_data/csse_covid_19_time_series/time_series_covid19_confirmed_global.csv) and the number of genomes from GISAID (by 8 August 2020). Only the genomes with collection date until 31 March 2020 were considered. When a given genome lacked the collection day date, only specifying the month of collection, it was assigned to the last day of the respective month. When the number of genomes on a given day was higher than the number of cases, the number of cases was considered for graphical representation. Final data on the assessment of genome sampling by country is provided in Supplementary material (Supplementary Data 2).

### Selecting a genomic background dataset

For the phylogenetic analyses, we downloaded full-length viral genome sequences from GISAID (https://www.gisaid.org/) on 6 August 2020 with collection dates before 1 April 2020 (Supplementary Data 2). For computational efficiency in the downstream operations, we analysed the A and B lineages separately. Multiple sequence alignments with a reference genome (MN908947.3) were performed using MAFFT v7.458 with parameter –addfragments^18^. Sequences with fewer than 75% unambiguous bases were excluded, as well as duplicate sequences defined as having identical nucleotide composition, collected on the same date and in the same country. The resulting dataset was trimmed at the 5′ and 3′ ends resulting in a multi-sequence alignment with 29,780 nucleotides. Sequences with date information only at the year-level were also excluded. This dataset was subjected to multiple iterations of phylogeny reconstruction using IQ-TREE multicore software version 1.6.12^19^ with parameters -m GTR+G, and exclusion of outlier sequences whose genetic divergence was incongruent with sampling date using TempEst software version 1.5.2^20^, resulting in 1632 and 22,124 sequences for the A and B datasets, respectively. GISAID acknowledgment table for the background dataset is provided as Supplementary Material (Supplementary Data 3).

### Subsampling strategy

The magnitude of the lineage B datasets prohibits a full Bayesian inference approach in a reasonable timeframe. To overcome this constraint, we used a subsampling strategy that removes sequences such that monophyletic clusters that consist entirely of sequences from a particular country are represented by a single sequence. The excess sequences in a country-specific monophyletic clade do not contribute any additional information to the between-country diffusion process we aim to infer^21^. This process resulted in a dataset with 13,489 sequences (B_CS). Despite the almost 40% downsampling in the B lineage dataset, its size is still excessive for timely computational inferences. To further address this issue, we have built a phylogeny using IQ-TREE, as described previously, and partitioned the tree into six monophyletic clades (B_CS1 through B_CS6). These clades were examined for outlier sequences whose genetic divergence and sampling date were incongruent using TempEst software version 1.5.2^20^. All data sets exhibited a positive correlation between genetic divergence and sampling time and appear to be suitable for phylogenetic molecular clock analysis^20^ (Supplementary Fig. 1).

### Bayesian evolutionary inference of SARS-CoV-2 detected in Portugal

A total of 1275 SARS-CoV-2 genome sequences (obtained from positive samples collected until 31 March 2020) from Portugal were analysed in this study (INSA’s collection, as of 23 July 2020; Supplementary Data 1). Our interest lies in estimating the viral evolutionary history and spatial diffusion process during the early epidemics in the country. Travel history data is of particular importance when analyzing low diversity data, such as that for SARS-CoV-2, using Bayesian joint inference of sequence and location traits because sharing the same location state can contribute to the phylogenetic clustering of taxa^13^. For each of the datasets (A, and B_CS1 through B_CS6), we performed a joint genealogical and phylogeographic inference of time-measured trees using Markov chain Monte Carlo (MCMC) sampling implemented in the Bayesian Evolutionary Analysis Sampling Trees (BEAST) package^22^. We applied a Hasegawa-Kishino-Yano 85 (HKY85) ^23^ substitution model with gamma-distributed rate variation among sites^24^. We used an uncorrelated lognormal relaxed molecular clock to account for evolutionary rate variation among lineages^25^ and specified an exponential growth coalescent prior in our analyses.

To integrate the travel history information obtained from (returning) travelers, we followed Lemey et al.^13^ and augmented the phylogeny with ancestral nodes that are associated with a location state (but not with a known sequence), and enforced the ancestral location at a point in the past of a lineage. We specified normal prior distributions on the travel times informed by an estimate of time of infection and truncated to be positive (back-in-time) relative to sampling date. Specifically, we used a period of 14 days (incubation period of 99% of patients^26^ where travel history information was collected for all recent movements), and a period between symptom onset and testing with an estimated mean of 4.70 days for the patients in the INSA cohort (estimated from data available for 717/1275 individuals), and a standard deviation of 4.06 days to incorporate the uncertainty on the period between symptom onset and testing. The location traits associated with taxa and with the ancestral nodes were modeled using a bidirectional asymmetric discrete diffusion process^27^. We ran and combined at least eight independent MCMC analyses for 50 million generations, sampling every 50,000th generation and removed 10% as chain burn-in. Stationarity and mixing were investigated using Tracer software version 1.7.1^28^, making sure that effective sample sizes for the continuous parameters were greater than 100. We used the high-performance computational capabilities of the Biowulf cluster at the National Institutes of Health (Bethesda, MD, USA) (http://biowulf.nih.gov) to perform these analyses. Portuguese clusters were assumed for phylogeographic summaries if their topology posterior probability was ≥0.001. If the excluded genomes had known travel history, they were re-integrated along with same-cluster sequences if those did not cluster in a clade-defining polytomy (recovered a total of 14 sequences, 7 of them with travel history). The location of the most recent common ancestor (MRCA) of Portuguese clusters is usually inferred as Portugal, thus we compared the estimated locations and times for the parent nodes of the MRCA (for simplicity, here on referred to as PMRCA) across Portuguese BEAST clades representing the origin and timing of seeding events into Portugal.

### Real-time data sharing of SARS-CoV-2 genetic diversity and geotemporal spread in Portugal

A website (https://insaflu.insa.pt/covid19) was launched on 28 March 2020 for real-time data sharing on SARS-CoV-2 genetic diversity and geotemporal spread in Portugal. This site gives access to “situation reports of the study and provides interactive data navigation using both Nextstrain (https://nextstrain.org/)^11^ and Microreact (https://microreact.org/)^12^ tools. As of 23 July 2020, genomic and phylogenetic analysis were performed using the SARS-CoV-2 Nextstrain pipeline version from 23 March 2020 (https://github.com/nextstrain/ncov), with slight modifications^15^. For data navigation, an IQ-TREE-derived^19^ phylogenetic tree enrolling the 1275 studied sequences, and the associated metadata, can be visualized interactively at https://microreact.org/project/cM6KURnU7rUpqdAnBq5DAf/a2d3840e.

### Ethical approval

Samples were obtained in the frame of the ongoing national SARS-CoV-2 genomic surveillance coordinated by the Portuguese National Institute of Health (INSA), being collected as part of the routine clinical care and laboratory procedures of the laboratories/hospitals (“Portuguese network for SARS-CoV-2 genomics”) collaborating in this system. This study was approved by the Ethical Committee (“Comissão de Ética para a Saúde”) of INSA, dismissing the need for individuals’ informed consent. Designations of all genome sequences are fully anonymized, and no identifying information of the associated patients is provided. Anonymized date of sample collection, date of illness onset and travel history were provided by laboratories and Regional and National Health Authorities.

### Reporting summary

Further information on research design is available in the Nature Research Reporting Summary linked to this article.

## Results

### Epidemiological trends and circulating diversity during the early COVID-19 pandemic in Portugal

**Fig. 1:**
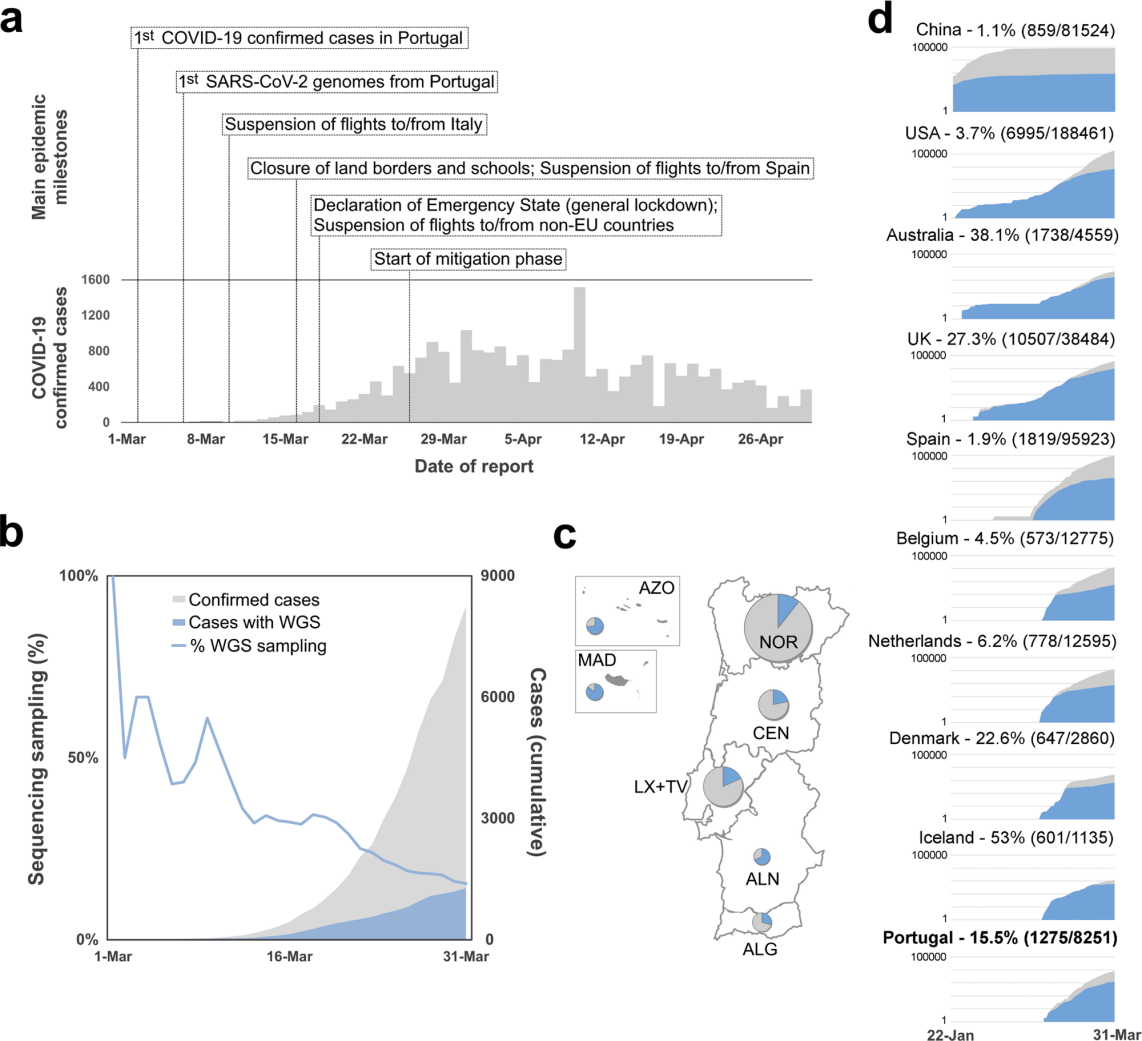
Overview of the COVID-19 confirmed cases and SARS-CoV-2 genome sequencing sampling in Portugal during the early phase of the pandemic. **a** Daily reported COVID-19 confirmed cases in Portugal and key milestones during the early phase of the pandemic (source: Directorate-General of Health, https://covid19.min-saude.pt/relatorio-de-situacao/). **b** Area plots (right *y*-axis) reflect the cumulative total number of COVID-19 confirmed cases (gray) and SARS-CoV-2 genome sequences (blue) reported/obtained in Portugal, until 31 March 2020. The blue line (left *y*-axis) displays the “sequencing sampling”, i.e. the proportion of confirmed cases with SARS-CoV-2 genome data during the same period. **c** SARS-CoV-2 sequencing sampling generated by the Health Administration region until 31 March 2020 (circles are proportional to the number of confirmed cases by Region, with the blue representing the proportion of samples with SARS-CoV-2 genome data). NOR Northern region, CEN Central region, LX+TV Lisbon and Tagus Valley region, ALN Alentejo, ALG Algarve, AZO Autonomous Region of Azores, MAD Autonomous Region of Madeira. **d** Area plots reflect the cumulative total number of COVID-19 confirmed cases (gray) and SARS-CoV-2 genome sequences (blue) reported by country between 22 January and 31 March 2020 for the top 10 countries with the highest number of genomes with collection date until 31 March 2020 (available on GISAID by 8th August 2020). A log10 scale *y*-axis was used for visualization purposes. Countries are ordered according to the date of the first reported COVID-19 case.

The first COVID-19 confirmed cases in Portugal were reported on 2 March 2020 after laboratory confirmation by the National Institute of Health (INSA) Doutor Ricardo Jorge. As the COVID-19 epidemic progressed in the country, INSA, as the National Reference Laboratory, gradually supported and validated the extension of the laboratory network throughout the country, where 37 laboratories were already set up to perform molecular testing of SARS-CoV-2 by March 31, 2020. With COVID-19 cases exponentially increasing in Portugal (Fig. 1a) and Europe (with alarming trends in Italy and Spain)^4^, Portugal adopted rigid public health measures, including suspension of flights from/to Italy (10 March 2020), closure of land borders and schools (16 March 2020), suspension of flights to non-EU countries and general lockdown (18 March 2020). INSA rapidly implemented and coordinated the nationwide genomic surveillance of SARS-CoV-2 (https://insaflu.insa.pt/covid19) with a particular focus on providing a comprehensive picture of the introductions, genetic diversification, and propagation of SARS-CoV-2 during the early-stage pandemic at a country scale. A total of 1275 SARS-CoV-2 positive samples collected in March were successfully subjected to virus genome sequencing (Supplementary Data 1), which corresponds to 15.5% (1275/8251) of all COVID-19 cases confirmed during the first month of the pandemic in Portugal (Fig. 1b). The viral genomic sequence sampling ranged from 10.7% (523/4910) in the Northern Health Administration region (the region with the highest number of confirmed cases) to 85.4% (41/48) in Madeira Archipelago (the region with the lowest number of confirmed cases) (Fig. 1c). Despite the delayed epidemic trajectory in comparison with other European countries, Portugal was among the countries generating the highest volumes of SARS-CoV-2 genomic data during the early COVID-19 pandemic (Fig. 1d and Supplementary Data 2).

The relative frequency trends of SARS-CoV-2 clades and lineages in Portugal during March 2020 (Supplementary Fig. 2) did not differ significantly from the scenario observed at the European level^5^. Most of the analyzed genomes (89.8%) belong to the 20A/G, 20B/GR, and 20C/GH clades (Nextstrain/GISAID nomenclatures), which display, among other genetic markers, the D614G amino acid replacement in the Spike protein. The SARS-CoV-2 G614 variant was most likely introduced in Europe by mid-January/early February 2020 and became dominant at a worldwide level^29^. Clades 19 A/L/V/O (dominant during early pandemic in China) and 19B/S (rare in Europe, with the notable exception of Spain)^5^ were found at the relative frequencies around 7.5% and 2.8% in March, respectively, and showed a decreasing frequency trend similarly to what has been observed at the global level. When applying the Pango lineages (cov-lineages.org) nomenclature, the main B lineage (roughly covering 19A/20A/20B/20C Nextstrain clades) was dominant in March, showing ample diversification into sublineages. Among these, it is highlighted the increased frequency of the B.1.1 sublineages (roughly corresponding to clade 20B/GR), as well as of the B.1, B.1.5, and B.1.91 lineages (all mostly including 20A/G virus). Of note, the B.1.91 sub-lineage, and part of its ancestor sublineage B.1, correspond to the Spike G614 + Y839 variant that massively disseminated in Portugal during the early epidemic (22% and 59% of the sampled genomes from the North and Center regions of Portugal by 30th April)^15^.

### Introductions and early spread of SARS-CoV-2 in Portugal

**Fig. 2:**
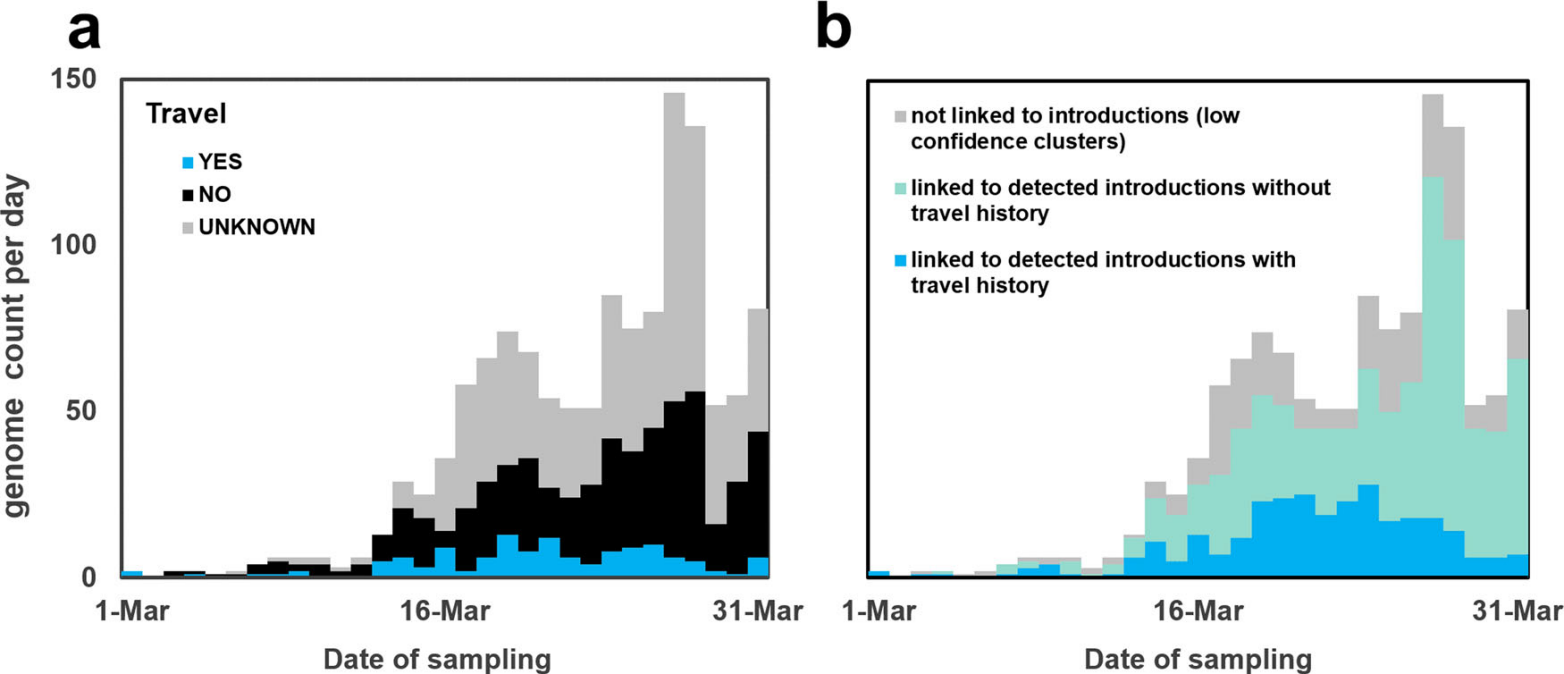
Overview of sequences linked to detected introductions, stratified by travel history data. **a** Histogram of COVID-19 confirmed cases with SARS-CoV-2 genome sequencing data stratified by travel history. **b** Histogram with sequence counts stratified by respective reconstruction linked with clades that include sequences from cases with (blue) or without (green) known travel history. Sequences belonging to BEAST clades with low topology posterior probability are shown in gray.

In order to assess the origins and measure the number of SARS-CoV-2 introductions in Portugal prior to 31 March 2020, we performed Bayesian phylodynamic analyses integrating the rich travel history data. The global TMRCA for the A lineage tree ranged between 11 November 2019–18 December 2019 (95% highest posterior density) and the global TMRCA for the B lineage trees ranged between 11 November 2019–3 February 2020 (95% highest posterior density). Travel history could be collected for 619 (48.5%) out of 1275 confirmed cases detected in March, with 128 (10.0%) reporting international travels within the potential incubation period (i.e., 14 days before clinical onset date) and 491 (38.5%) reporting no travels within the same period (Fig. 2a).

**Fig. 3:**
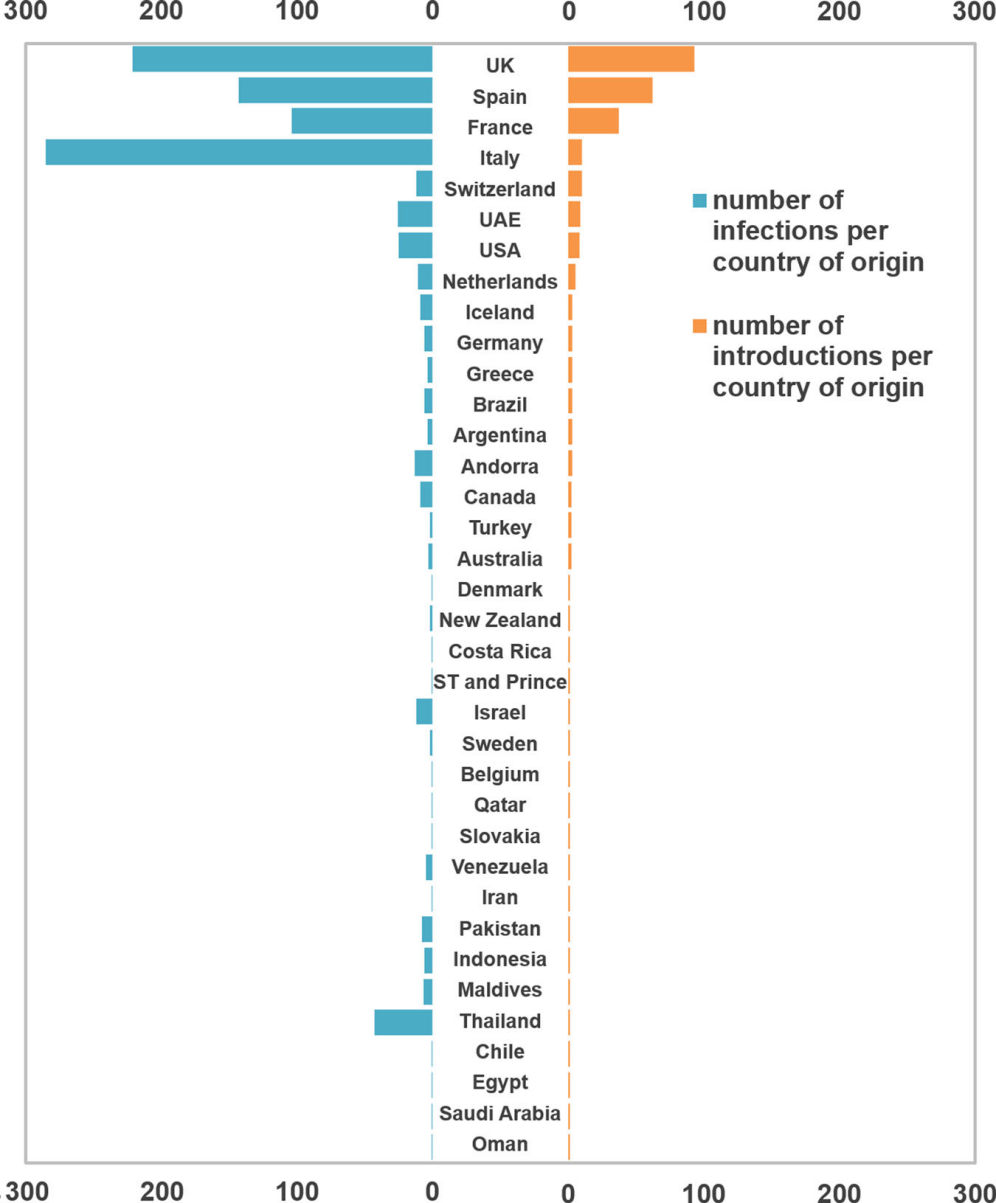
Number and size of SARS-CoV-2 introductions per country. Bar plots represent the number of introductions by country of origin (orange) and respective total number of cases (blue), inferences are derived from the phylodynamic reconstruction.

**Fig. 4:**
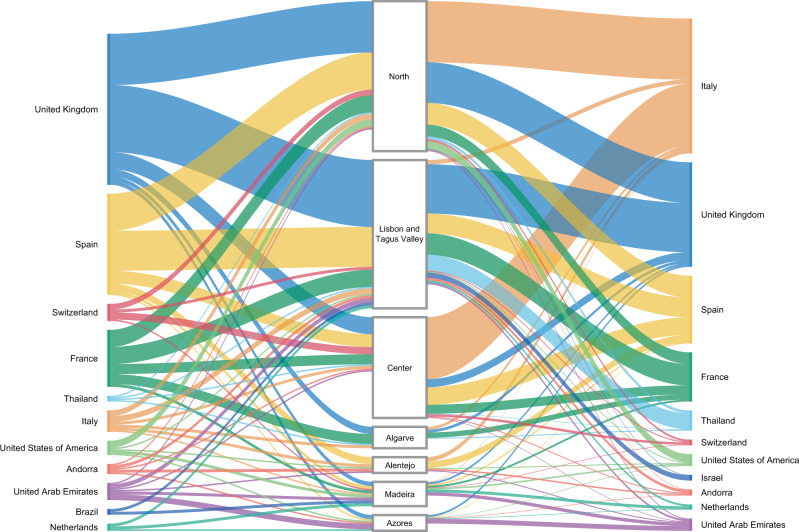
Sankey plots summarizing the viral flow into Portuguese Health Administration regions. Left. The plot shows the relative number of transitions between countries of origin and different regions in Portugal. For summaries that show all transitions to and from all connected locations, we refer to the Supplementary Data 4. Right. The plot shows the relative number of infections generated by introductions from countries of origin and different regions in Portugal. For summaries that show the size of all introductions to and from all connected locations, we refer to the Supplementary Data 5. For both panels, for graphics simplicity, we present the eleven countries linked with most introductions (left) and relative number of infections generated (right), estimated across phylogenies inferred for the whole dataset, i.e., including both Pango lineages A and B (thin – low proportion of number/size of viral introductions attributed to this source; thick – high proportion of number/size of viral introductions attributed to this source). We note that there is no temporal order for the transitions involved.

**Fig. 5:**
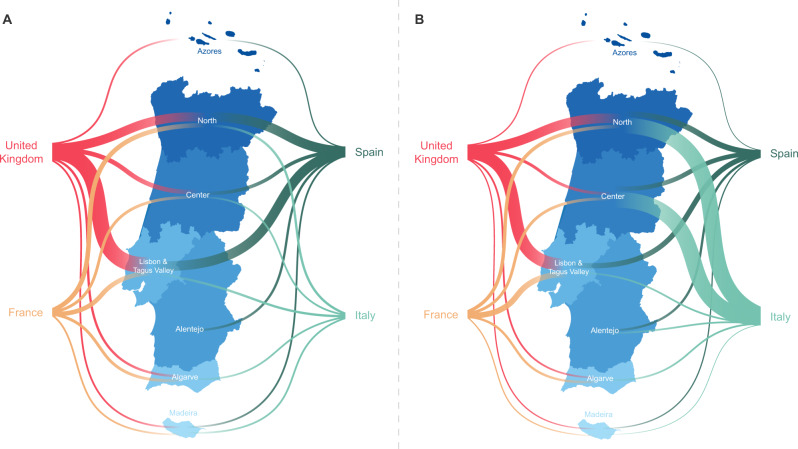
Map summarizing the viral flow into Portuguese Health Administration regions. **A** The plot shows the relative number of transitions between the four countries of origin linked with most introductions to different regions in Portugal. For summaries that show all transitions to and from all connected locations, we refer to the Supplementary Data 4. **B** The plot shows the relative number of infections generated by introductions from the four countries of origin linked with most relative number of infections generated in different regions in Portugal. For summaries that show the size of all introductions to and from all connected locations, we refer to the Supplementary Data 5. For a broader overview of the 11 countries linked with most introductions and relative number of infections generated, we also refer to Fig. 4. For both panels, introductions (left) and relative number of infections generated (right) were estimated across phylogenies inferred for the whole dataset, i.e., including both Pango lineages A and B (thin – low proportion of number/size of viral introductions attributed to this source; thick – high proportion of number/size of viral introductions attributed to this source). We note that there is no temporal order for the transitions involved.

Overall, we detected 277 clades (16 from lineage A and 261 from lineage B) representing SARS-CoV-2 introductions in Portugal until 31 March 2020, involving a total of 979 out of the 1275 sequences analyzed. Of these, 296/979 genomes belong to clades that include sequences from cases with known travel history (Fig. 3b). Overall, the phylogeographic reconstruction revealed that UK, France, and Spain seeded 69% of all introductions into Portugal (Fig. 3), mostly to the Lisbon and Tagus Valley region, which was estimated to have received ~30% of all introductions, followed by the North (27%), Center (10%), Algarve (4%), Azores (2%), Alentejo (2%), and Madeira (1%) regions (Figs. 4 and 5A and Supplementary Data 4).

We also examined the number of infections generated by introductions from different countries (Figs. 3, 4, and 5B and Supplementary Data 5). Interestingly, despite the UK, France, and Spain having seeded the majority of introductions, at least one seeding event linked to travel history from Italy generated the largest outbreak recorded in the early stages of the COVID-19 epidemic in Portugal (cfr. below)^15^. We noticed a similar pattern for an introduction from Thailand, however, this event had a low supported topology posterior probability (Figs. 4 and 5B and Supplementary Data 5).

The spatial analysis reconstructed 16 introductions of lineage A into Portugal encompassing 33 Portuguese sequences. The majority of this lineage viral flow was seeded by Spain (69%), with 41% of all of these being introduced into Lisbon & Tagus Valley, 12% into the Center and Alentejo regions, respectively, and 6% into the North region. The remainder of all lineage A seeding events were estimated to have originated from Australia, France, Italy, Oman, Saudi Arabia and were mostly captured by the ability to integrate travel history data into the phylogeographic reconstruction (Supplementary Fig. 3 and Supplementary Data 6). The introductions resulted in transmission clusters of varying size, with the largest, seeded by Spain, generating a transmission chain of at least 7 cases in Alentejo.

A similar phylogeographic analysis for lineage B resulted in the reconstruction of 261 introductions covering 946 Portuguese genomes. The majority of lineage B introductions was seeded by the United Kingdom (UK) (36%), Spain (20%), and France (14%). The introductions resulted in transmission clusters of varying sizes. The largest cluster was seeded by Italy and generated a transmission chain of at least 252 cases in the North and Center regions. Switzerland (4%), United Arab Emirates (3%), United States of America (3%), Netherlands (2%) also contributed to the remainder of lineage B introductions and establishment in the country (Supplementary Fig. 4 and Supplementary Data 7).

**Fig. 6:**
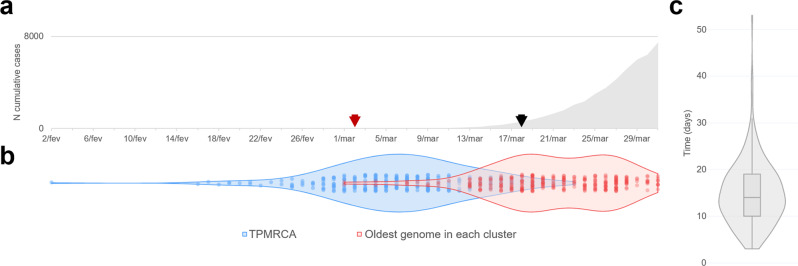
Cryptic transmission of SARS-CoV-2 in Portugal revealed by genomic epidemiology. **a** Number of cumulative cases over time (red and black arrows indicate the date of notification of the first COVID-19 case detected in Portugal and the start of the general lockdown, respectively) **b** Violin plots represent the date of sample collection of the oldest genome in a Portuguese clade (red) and the times for the parent nodes of the most recent common ancestors (TPMRCA in blue) for each of the 277 inferred introductions. **c** Violin and box plots depict the time lag between the introduction and the first surveilled genome for all 277 clusters.

These analyses revealed a median time lag of 14 days (range = 3 and 53 days; Fig. 6) between the time to the PMRCA (TPMRCA; see Methods section for details), representing the earliest an introduction could have occurred, and surveilled genomes. In particular, the TPMRCA of most clades (~58%) occurred between the last week of February and the first week of March (Supplementary Data 8). The temporal reconstruction estimated the earliest introduction of lineage A into Portugal to have occurred as early as 7 March 2020 [95% Highest Posterior Density (HPD): 4 March 2020–10 March 2020], from Spain to the Lisbon & Tagus Valley region. The earliest seeding event for lineage B was estimated to have taken place on 2 February 2020 (95% HPD: 30 January 2020–5 February 2020) via the United Kingdom into the North region. Of note, 108 out of the 277 introductions were seeded by Nextstrain 20B clade viruses. Because of both the high abundance of 20B sequences in the dataset and the low genetic diversity within this clade at the time (leading to polytomy-rich topologies), the TPMRCAs of these introductions might be less accurate in representing the importation date into Portugal. Notwithstanding, our data generally indicates that most introductions were seeded before main public health interventions and when the cumulative number of detected cases was still very low (Figs. 1 and 6), suggesting a period of several weeks where undetected transmission likely occurred (Fig. 6).

## Discussion

The present study provides a comprehensive description of the early establishment of the COVID-19 pandemic in Portugal based on a genomic epidemiology and phylodynamics approach. We detected multiple independent SARS-CoV-2 introductions, mostly from European countries (namely the UK, Spain, France, Italy, and Switzerland), which was broadly consistent with the available travel history data, as well as with the countries with most frequent flight connectivity with Portugal (INE, 2020) and/or with the highest number of Portuguese immigrants^30,31^.

The genomic surveillance efforts in Portugal culminated with the generation of 1275 SARS-CoV-2 whole genomes, which represents 15.5% of all confirmed cases in March, making Portugal the country generating the 5th highest number of SARS-CoV-2 genomic sequences during the early COVID-19 pandemic. In particular, our data uncovered the importation of extensive SARS-CoV-2 genomic diversity during the early epidemic in Portugal, as supported by the identification of at least 277 seeding events, covering all Nextstrain/GISAID clades and dozens of Pango lineages. Despite the vast genetic diversity introduced, 32% of the 1275 analyzed sequences could be linked to just ten out of the 277 detected introductions. In particular, a single introduction (from Italy) of a Spike Y839 variant represented about 20% of all sampled genomes collected until the end of March^15^. Moreover, 56% (155/277) of BEAST clades involve one single sequence (singletons), thus suggesting that most introductions have not seeded substantial local transmission.

In general, these data suggest that the implemented preventive and early control measures have been successful in mitigating the establishment of community transmission from most SARS-CoV-2 independent introductions. On the other hand, it also highlights that the few introductions not captured by public health surveillance and control can still seed large community transmission events and give rise to a significant number of cases. This underlies the challenges in defining a public health strategy aimed at preventing sustained community transmission while maintaining open borders and global connectivity. Portugal presents a geopolitical and demographic context involving high circulation of both migrant workers and tourists, and close international networks with multiple countries from different latitudes (particularly from Europe, South America, and Africa). More stringent border restrictions, including flight and land borders closure, were implemented after March 10, when 74% (205/277) of the detected introductions were estimated to have already occurred. Despite the delay in the epidemic start in Portugal in comparison with other European countries, which certainly contributed to a more favorable situation during the pandemic’s first epidemic wave, the earlier implementation of measures (close borders, travelers testing, etc.) could have largely minimized the number of introductions and subsequent virus expansion.

The detection lag observed between the estimated time of the most recent common ancestor and the collection date of the earliest sample of each BEAST clade denotes that cryptic transmission might have occurred to some extent. Although most introductions were estimated to have occurred during the last week of February and, especially, during the first week of March 2020, SARS-CoV-2 was silently circulating in Portugal a few weeks before the first confirmed local cases on 2 March 2020. For instance, 12 out of 19 sequences collected during the first week of the epidemic were obtained from patients with no travel history, including patients linked to the introduction leading to the highest number of cases during the first wave. These results are reasonable epidemiologically given the period needed for the diagnostics infrastructure to be set up. Also, the period of cryptic transmission reported in this study for Portugal is within the estimated for other countries during the early pandemic period^32,33^.

Phylodynamic modeling relies on the accumulation of genetic diversity over time for the estimation of the evolutionary rate and timing of other relevant events. Being a recently emerged pathogen with a relatively low substitution rate (in comparison with other RNA viruses) and a large genome, it is challenging to study the early dynamics of SARS-CoV-2, as a large proportion of sequences collected across time and space are very closely related genetically, thus complicating the reconstruction of the phylogenetic topology. To address this, we opted to focus on a subset of the results with higher topological support. Furthermore, due to the computational burden required for phylodynamic analysis, it is unfeasible to include all available genomic sequences. One limitation of this and other studies trying to infer the origin of introductions is the sequencing sampling bias by country. For this reason, we resorted to filtering and subsampling strategies that aim at reducing the number of genomes that do not significantly contribute to the evolutionary or phylogeographic reconstructions. Despite being less pronounced, there were still discrepancies in the number of genomes included across countries. For instance, it is very likely that the number of introductions via the UK (the country generating the highest volume of sequences) is overestimated, while the number of introductions from countries with none or few available genome sequences is underestimated. However, the integration of travel history during the phylodynamic inferences allows for a more accurate reconstruction of the spatial pathways from both over and undersampled locations. Additionally, using this recently developed model^13^ provides insight into the genetic diversity circulating in countries for which genomic surveillance is still lacking. This makes the present work among the first to apply this novel model, which allowed us to gain insight into the early circulating diversity in countries like Saint Thomas and Prince, for which (as of 15 February 2021) there are no sequences available, and other undersampled countries such as the United Arab Emirates and Qatar.

Overall, our findings highlight the use of genomic data to trace the introduction and spread of an emerging virus, showing the need of systematic, continuous, and geographically representative genomic surveillance to detect and monitor the emergence and dissemination of biologically and/or epidemiologically relevant variants. Together with open data sharing, the timely generation of SARS-CoV-2 genomic data has been shown to be an invaluable tool to guide national and international public health authorities towards the identification and control of highly transmissible and/or immune evading variants^34^.

In this context, by laying the foundation of the genomic epidemiology of SARS-CoV-2 in Portugal involving an unprecedented collaborative effort at national and international levels, this work and concomitant capacity building, was pivotal for Portugal’s response to the current and upcoming needs of genomic-informed surveillance and epidemiology of COVID-19, as strongly recommended by ECDC and WHO^10,35,36^.

## Supplementary information

Supplementary Material

Description of Additional Supplementary Files

Supplementary Data 1

Supplementary Data 2

Supplementary Data 3

Supplementary Data 4

Supplementary Data 5

Supplementary Data 6

Supplementary Data 7

Supplementary Data 8

Reporting Summary

## Data Availability

SARS-CoV-2 genome sequences generated in this study were uploaded to the GISAID database (https://www.gisaid.org/). Accession numbers can be found in Supplementary material (Supplementary Data 1). Source data for the main figures in the manuscript can be accessed as: (i) Fig. 1. Daily reported COVID-19 confirmed cases in Portugal (https://covid19.min-saude.pt/relatorio-de-situacao/) (Fig. 1a–c); SARS-CoV-2 genome sequences reported/obtained in Portugal, until 31 March 2020 (Supplementary Data 1; https://microreact.org/project/cM6KURnU7rUpqdAnBq5DAf/a2d3840e) (Fig. 1b, c); Cumulative total number of COVID-19 confirmed cases and SARS-CoV-2 genome sequences reported by country between January 22 and March 31 (Supplementary Data 2 and 3; https://github.com/CSSEGISandData/COVID-19/blob/master/csse_covid_19_data/csse_covid_19_time_series/time_series_covid19_confirmed_global.csv); (ii) Fig. 2. Sequences linked to detected introductions, stratified by travel history data (https://microreact.org/project/cM6KURnU7rUpqdAnBq5DAf/a2d3840e); (iii) Fig. 3. Number and size of SARS-CoV-2 introductions per country (https://microreact.org/project/cM6KURnU7rUpqdAnBq5DAf/a2d3840e and Supplementary Data 8); Figs. 4e and 5. Relative number of transitions between countries of origin and different regions in Portugal (Supplementary Data 4; (https://microreact.org/project/cM6KURnU7rUpqdAnBq5DAf/a2d3840e) and relative number of infections generated by introductions from countries of origin and different regions in Portugal. (Supplementary Data 5; (https://microreact.org/project/cM6KURnU7rUpqdAnBq5DAf/a2d3840e); Fig. 6. Daily reported COVID-19 confirmed cases in Portugal (https://covid19.min-saude.pt/relatorio-de-situacao/) and size, mean date estimates (and respective 95% highest posterior density intervals) of parent nodes of the MRCA and the earliest date of sample collection for each BEAST cluster (Supplementary Data 8).
