# Supplementary material for: SARS-CoV-2 introductions and early dynamics of the epidemic in Portugal
Source: Commun Med (Lond). 2022 Jan 28;2:10. doi: 10.1038/s43856-022-00072-0 (PMC9053228; doi:10.1038/s43856-022-00072-0)
Supplement: Supplementary file 1 — Supplementary Material [file 43856_2022_72_MOESM1_ESM.pdf]

Supplementary Material

Supplementary Figures

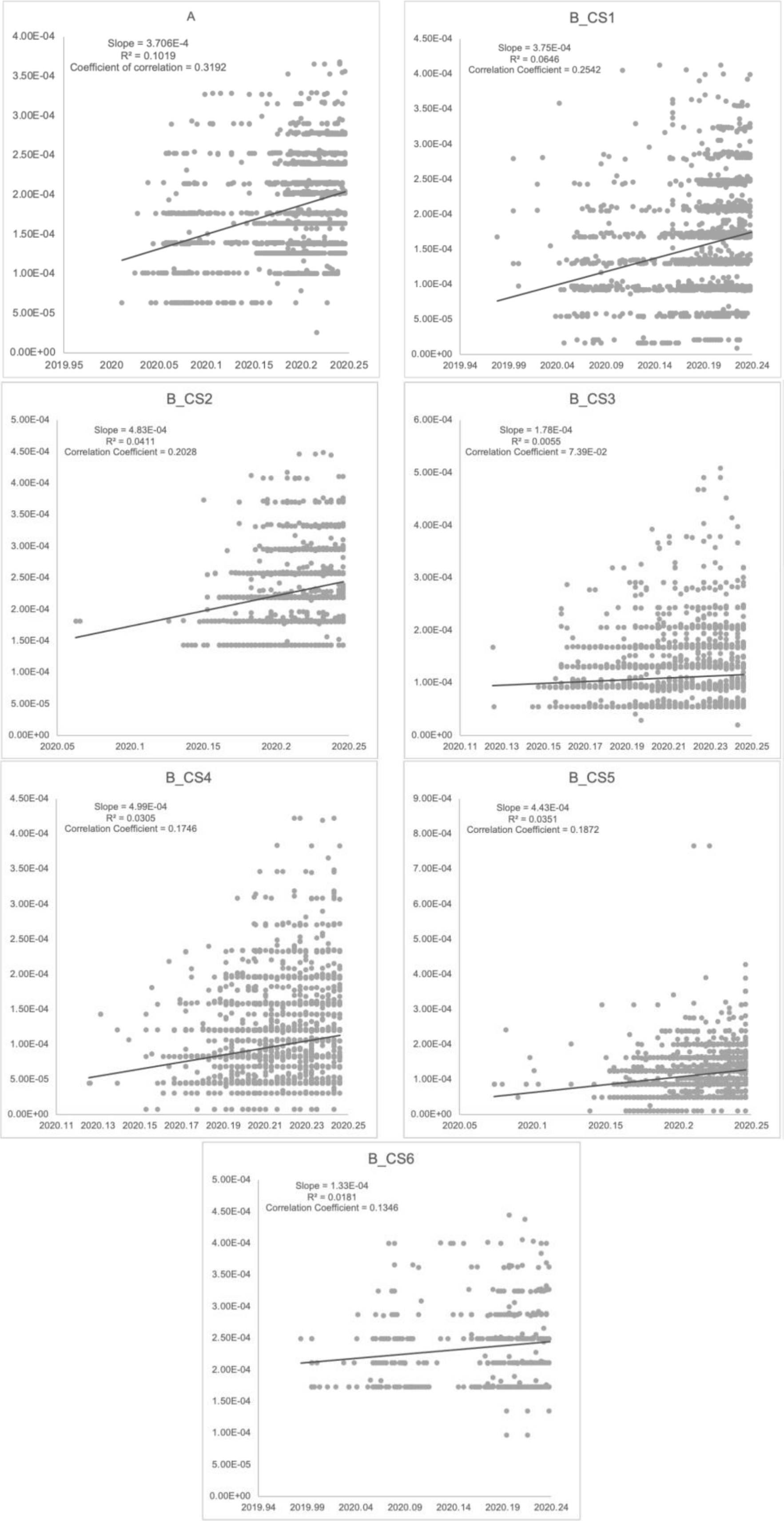

Supplementary Figure 1. Root-to-tip regression analyses. Plots of the root-to-tip genetic distance against sampling time are shown for phylogenies estimated from alignments of datasets A, and BCS_1 to BCS_6.

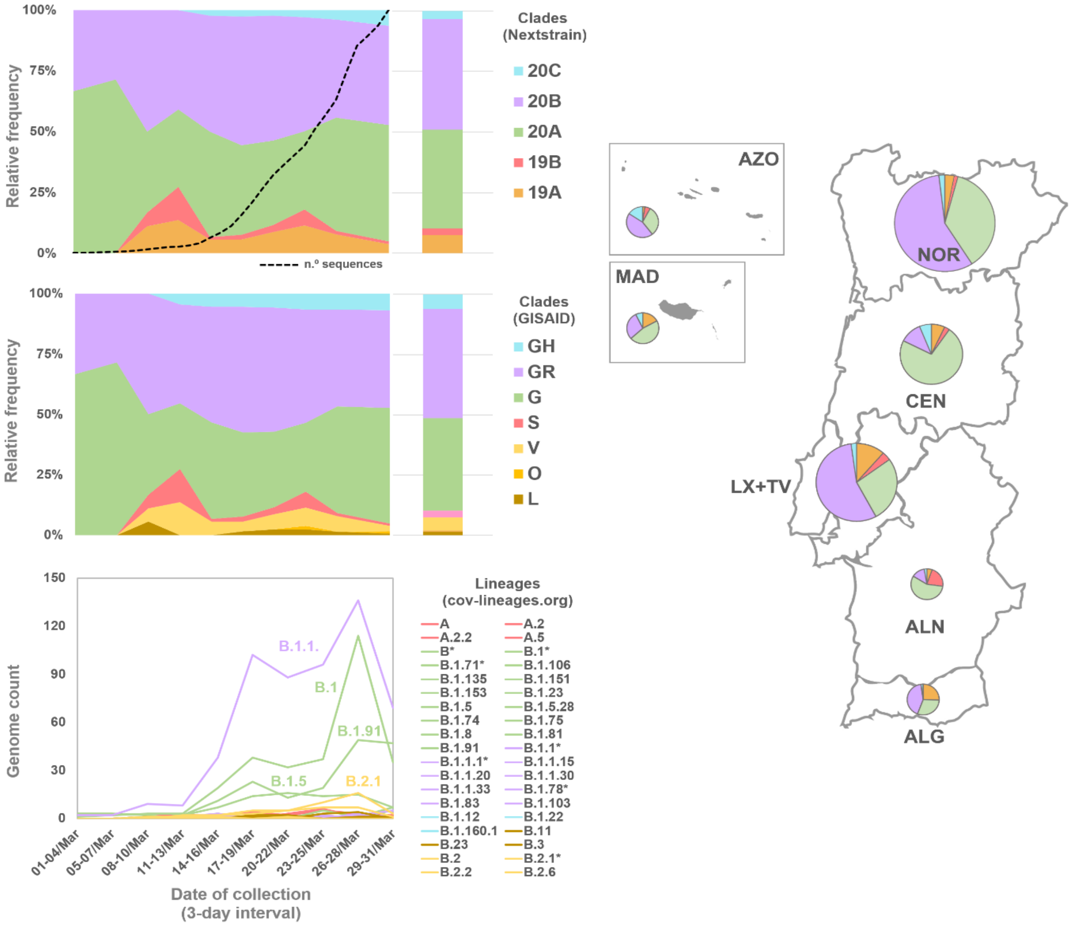

Supplementary Figure 2. Frequency trends of SARS-CoV-2 clade and lineages in Portugal during March 2020 (n = 1,275), according to the following nomenclature approaches: Nextstrain (https://clades.nextstrain.org/); GISAID (https://www.gisaid.org/), and Phylogenetic Assignment of Named Global Outbreak LINeages (cov-lineages.org) (https://pangolin.cog-uk.io/). Area plots reflect the Nextstrain/GISAID clade relative frequency by collection date (using 3-day interval with exception 1-4 March interval), while bar plots reflect the global relative frequency observed in March 2020. For the sake of graphical simplicity, the trajectory of the cov-lineages is shown as genome count instead of relative frequency. Pango lineages are colored according to the corresponding GISAID clades (when 100% congruence). For incongruent cases (labeled by an asterisk), the color reflects the most abundant GISAID clade within a given Pango lineage. The dashed line in the Nextstrain classification panel reflects the cumulative number of COVID-19 confirmed cases as shown in Figure 1. The map shows the relative frequency of Nextstrain clades by Health Administration region (circles are proportional to the number of confirmed cases by region) in March 2020. Classification is detailed in Supplementary Table 1. The phylogeny, clade/lineage classification and geotemporal distribution can be visualized interactively at https://microreact.org/project/cM6KURnU7rUpqdAnBq5DAf/a2d3840e.

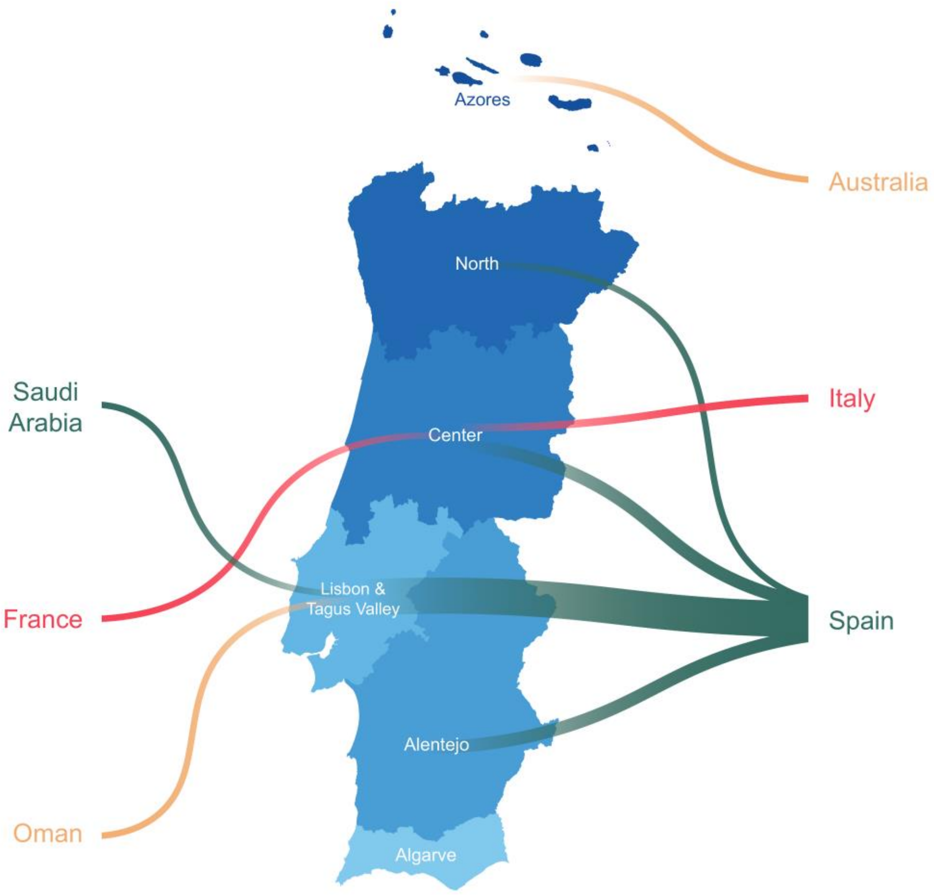

Supplementary Figure 3. Map summarizing the viral gene flow of Pango lineage A into Portugal Health Administration Regions. The map shows the relative number of transitions between countries of origin and different regions in Portugal. Specifically, the line width represents the relative proportion of viruses seeded into a given Portuguese Health Administration Region by various countries, estimated across phylogenies inferred for Pango lineage A (thin – low proportion of viral introductions attributed to this source; thick – high proportion of viral introductions attributed to this source). We note that there is no temporal order for the transitions involved. For summaries that show all transitions to and from all connected locations for lineage A viruses, we refer to the Supplementary Table 6.

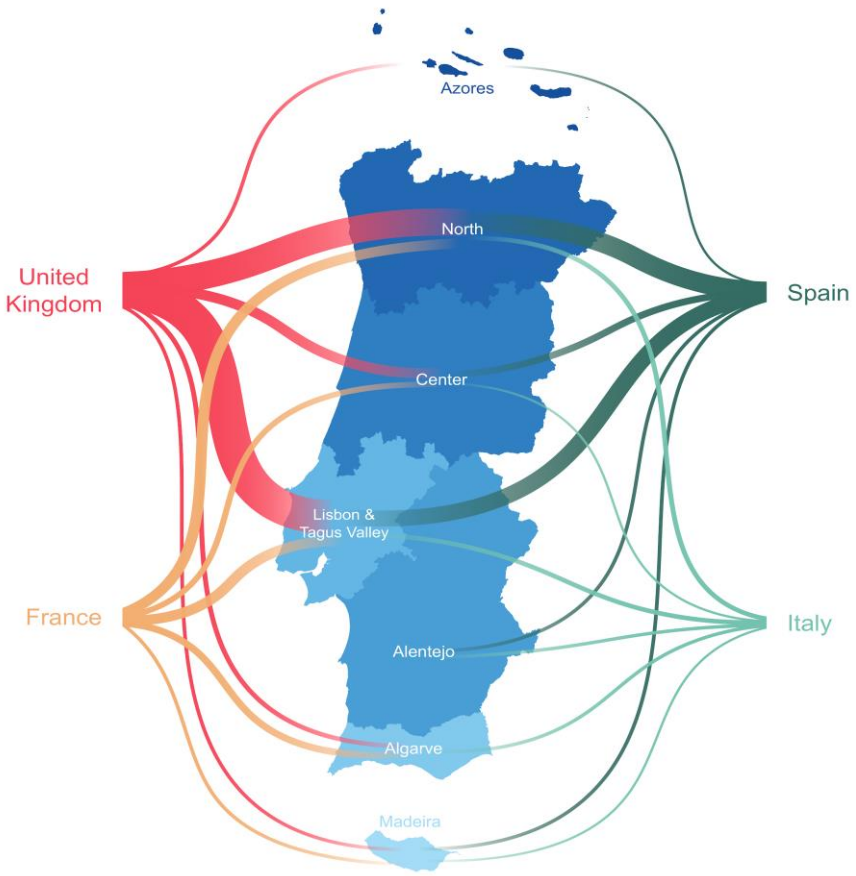

Supplementary Figure 4. Map summarizing the viral gene flow of Pango lineage B into Portugal Health Administration Regions. The map shows the relative number of transitions between countries of origin and different regions in Portugal. Specifically, the line width represents the relative proportion (>0.9%) of viruses seeded into a given Portuguese Health Administration Region by various countries, estimated across phylogenies inferred for Pango lineage B (thin – low proportion of viral introductions attributed to this source; thick – high proportion of viral introductions attributed to this source). We note that there is no temporal order for the transitions involved. For summaries that show all transitions to and from all connected locations for lineage B viruses, we refer to the Supplementary Table 7.
