# Supplementary material for: SARS-CoV-2 introductions and early dynamics of the epidemic in Portugal
Source: Commun Med (Lond). 2022 Jan 28;2:10. doi: 10.1038/s43856-022-00072-0 (PMC9053228; doi:10.1038/s43856-022-00072-0)
Supplement: Supplementary file 2 — Description of Additional Supplementary Files [file 43856_2022_72_MOESM2_ESM.pdf]

Description of Additional Supplementary Files

File Name: Supplementary Data 1

Description: List of SARS-CoV-2 genome sequences (and respective Clade/lineage classification) from Portugal used in this study.

File Name: Supplementary Data 2

Description: Number of cumulative cases of COVID-19 by 31 March 2020, by country, and respective number of available SARS-CoV-2 genomes with collection date until 31 March (and available on GISAID as of 8 August 2020). Countries are shown by descending order of number of genomes and only countries with at least 30 COVID-19 reported cases and more than 2 genomes available are represented. Countries highlighted in bold are represented in Figure 1D.

File Name: Supplementary Data 3

Description: GISAID acknowledgment table for the background dataset. We acknowledge the original and submitting laboratories that generated the full-length viral genome sequences downloaded from GISAID (https://www.gisaid.org/) on August 6, 2020 with collection dates before April 1, 2020.

File Name: Supplementary Data 4

Description: Heatmap with relative number of transitions between countries of origin and different regions in Portugal

File Name: Supplementary Data 5

Description: Heatmap with relative number of infections generated by introductions from countries of origin and different regions in Portugal.

File Name: Supplementary Data 6

Description: Heatmap with relative number of transitions between countries of origin and different regions in Portugal for viruses belonging to Pango lineage A.

File Name: Supplementary Data 7

Description: Heatmap with relative number of transitions between countries of origin and different regions in Portugal for viruses belonging to Pango lineage B.

File Name: Supplementary Data 8

Description: Size, mean date estimates (and respective 95% highest posterior density intervals) of parent nodes of the MRCA and the earliest date of sample collection for each BEAST cluster.
