# Supplementary material for: SARS-CoV-2 introductions and early dynamics of the epidemic in Portugal
Source: Commun Med (Lond). 2022 Jan 28;2:10. doi: 10.1038/s43856-022-00072-0 (PMC9053228; doi:10.1038/s43856-022-00072-0)
Supplement: Supplementary file 11 — Reporting Summary [file 43856_2022_72_MOESM11_ESM.pdf]

nature research

Corresponding author(s): JP Gomes

Last updated by author(s): Dec 15, 2021

Reporting Summary

Nature Research wishes to improve the reproducibility of the work that we publish. This form provides structure for consistency and transparency in reporting. For further information on Nature Research policies, see our Editorial Policies and the Editorial Policy Checklist.

Statistics

For all statistical analyses, confirm that the following items are present in the figure legend, table legend, main text, or Methods section.

n/a	Confirmed	
☐	☒	The exact sample size (n) for each experimental group/condition, given as a discrete number and unit of measurement
☐	☒	A statement on whether measurements were taken from distinct samples or whether the same sample was measured repeatedly
☐	☒	The statistical test(s) used AND whether they are one- or two-sided<br>*Only common tests should be described solely by name; describe more complex techniques in the Methods section.*
☐	☒	A description of all covariates tested
☒	☐	A description of any assumptions or corrections, such as tests of normality and adjustment for multiple comparisons
☒	☐	A full description of the statistical parameters including central tendency (e.g. means) or other basic estimates (e.g. regression coefficient) AND variation (e.g. standard deviation) or associated estimates of uncertainty (e.g. confidence intervals)
☒	☐	For null hypothesis testing, the test statistic (e.g. F, t, r) with confidence intervals, effect sizes, degrees of freedom and P value noted<br>*Give P values as exact values whenever suitable.*
☐	☒	For Bayesian analysis, information on the choice of priors and Markov chain Monte Carlo settings
☒	☐	For hierarchical and complex designs, identification of the appropriate level for tests and full reporting of outcomes
☒	☐	Estimates of effect sizes (e.g. Cohen's d, Pearson's r), indicating how they were calculated

Our web collection on statistics for biologists contains articles on many of the points above.

Software and code

Policy information about availability of computer code

Data collection	Genetic data was either generated or retrieved from the GISAID dataset.
Data analysis	Genome assembly and curation was performed using INSaFLU (Trimmomatic v0.27, Snippy v3.2) and Clade and lineage classification was performed with Nextstrain (as of 9 November 2020), GISAID (as of 23 July 2020) and Phylogenetic Assignment of Named Global Outbreak LINeages (as of 16 October 2020). The genetic datasets were subsampled using a previously published R script. The genetic datasets were curated using MAFFT v7, AliView v.1.27, IQTree v2 and TempEst v1.5.3. Phylodynamic analysis were performed and interpreted using BEAST v1.10.5, Tracer v1.7.1 , FigTree v1.4.4.

For manuscripts utilizing custom algorithms or software that are central to the research but not yet described in published literature, software must be made available to editors and reviewers. We strongly encourage code deposition in a community repository (e.g. GitHub). See the Nature Research guidelines for submitting code & software for further information.

Data

Policy information about availability of data

All manuscripts must include a data availability statement. This statement should provide the following information, where applicable:

- Accession codes, unique identifiers, or web links for publicly available datasets
- A list of figures that have associated raw data
- A description of any restrictions on data availability

SARS-CoV-2 genome sequences generated in this study were uploaded to the GISAID database (https://www.gisaid.org/). Accession numbers can be found in Supplementary material.

Field-specific reporting

Please select the one below that is the best fit for your research. If you are not sure, read the appropriate sections before making your selection.

☐ Life sciences ☐ Behavioural & social sciences ☒ Ecological, evolutionary & environmental sciences

For a reference copy of the document with all sections, see nature.com/documents/nr-reporting-summary-flat.pdf

Ecological, evolutionary & environmental sciences study design

All studies must disclose on these points even when the disclosure is negative.

Study description	Genomic epidemiology of SARS-CoV-2 in Portugal
Research sample	SARS-CoV-2 positive samples (either clinical specimens or RNA) were provided to the National Institute of Health for viral genomic analysis by a nationwide network, consisting of more than 50 laboratories in Portugal.
Sampling strategy	All SARS-CoV-2 positive samples collected in Portugal until 31 March 2020.
Data collection	SARS-CoV-2 positive samples (either clinical specimens or RNA) were sent to the National Reference Laboratory hosted at the National Institute of Health, together with minimal descriptive data, following standard procedures.
Timing and spatial scale	Nationwide sample collection, ranging from 01 March 2020 to 31 March 2020
Data exclusions	Data was excluded when viral genome sequencing was not successful.
Reproducibility	All sequence data is available, so our methods detailed description allows the reproducibility of our findings.
Randomization	This is not relevant as this study aimed to study all SARS-CoV-2 positive samples collected in Portugal until 31 March 2020.
Blinding	Sample descriptive data were anonymized and the procedures were approved by the Ethical Committee ("Comissão de Ética para a Saúde") of the Portuguese National Institute of Health.

Did the study involve field work? ☐ Yes ☒ No

Reporting for specific materials, systems and methods

We require information from authors about some types of materials, experimental systems and methods used in many studies. Here, indicate whether each material, system or method listed is relevant to your study. If you are not sure if a list item applies to your research, read the appropriate section before selecting a response.

Materials & experimental systems

n/a	Involved in the study
☒	☐ Antibodies
☒	☐ Eukaryotic cell lines
☒	☐ Palaeontology and archaeology
☒	☐ Animals and other organisms
☒	☐ Human research participants
☒	☐ Clinical data
☒	☐ Dual use research of concern

Methods

n/a	Involved in the study
☒	☐ ChIP-seq
☒	☐ Flow cytometry
☒	☐ MRI-based neuroimaging
